# Variable participation of knowledge users in cancer health services research: results of a multiple case study

**DOI:** 10.1186/s12874-018-0593-8

**Published:** 2018-11-22

**Authors:** Mary Ann O’Brien, Andrea Carson, Lisa Barbera, Melissa C. Brouwers, Craig C. Earle, Ian D. Graham, Nicole Mittmann, Eva Grunfeld

**Affiliations:** 1 Department of Family and Community Medicine, University of Toronto, 500 University Avenue, Fifth Floor, Toronto, ON M5G 1V7 Canada; 2 Social and Behavioural Health Sciences, Dalla Lana School of Public Health, University of Toronto, Toronto, ON Canada; 3 Tom Baker Cancer Centre, Calgary, AB Canada; 4 University of Calgary, Calgary, AB Canada; 5 Institute for Clinical Evaluative Sciences, Toronto, ON Canada; 6 School of Epidemiology and Public Health, Faculty of Medicine, University of Ottawa, Ottawa, ON Canada; 7 Ontario Institute for Cancer Research, Toronto, ON Canada; 8 School of Epidemiology and Public Health, University of Ottawa, Ottawa, ON Canada; 9 Ottawa Hospital Research Institute, Ottawa, ON Canada; 10 Cancer Care Ontario, Toronto, ON Canada; 11 Sunnybrook Research Institute, Toronto, ON Canada; 12 Odette Cancer Centre, Sunnybrook Health Sciences Centre, Toronto, ON Canada; 13 Department of Oncology, McMaster University, Hamilton, ON Canada

**Keywords:** Cancer health services research, Integrated knowledge translation, Knowledge users, Qualitative research, Case study

## Abstract

**Background:**

Integrated knowledge translation (IKT) is a research approach in which knowledge users (KUs) co-produce research. The rationale for IKT is that it leads to research that is more relevant and useful to KUs, thereby accelerating uptake of findings. The aim of the current study was to evaluate IKT activities within a cancer health services research network in Ontario, Canada.

**Methods:**

An embedded multiple case study design was used. The cases were 5 individual studies within an overarching cancer health services research network. These studies focused on one of the following topics: case costing of cancer treatment, lung cancer surgery policy analysis, patient and provider-reported outcomes, colorectal cancer screening, and a team approach to women’s survivorship. We conducted document reviews and held semi-structured interviews with researchers, KUs, and other stakeholders within a cancer system organization. The analysis examined patterns across and within cases.

**Results:**

Researchers and their respective knowledge users from 4 of the 5 cases agreed to participate. Eighteen individuals from 4 cases were interviewed. In 3 of 4 cases, there were mismatched expectations between researchers and KUs regarding KU role; participants recommended that expectations be made explicit from the beginning of the collaboration. KUs perceived that frequent KU turnover may have affected both KU engagement and the uptake of study results within the organization. Researchers and KUs found that sharing research results was challenging because the organization lacked a framework for knowledge translation. Uptake of research findings appeared to be related to the researcher having an embedded role in the cancer system organization and/or close alignment of the study with organizational priorities. Document reviews found evidence of planned IKT strategies in 3 of 4 cases; however, actual KU role/engagement on research teams was variable.

**Conclusions:**

Barriers to KU co-production of cancer health services research include mismatched expectations of KU role and frequent KU turnover. When a research study directly aligns with organizational priorities, it appears more likely that results will be considered in programming. Research teams that take an IKT approach should consider specific strategies to address barriers to KU engagement.

## Introduction

A central goal of health research is to generate new knowledge and make useful discoveries that will benefit the population of interest. While important, knowledge generation and innovation alone may not lead to implementation of evidence-based interventions in clinical practice or policy. The failure to implement research findings has been identified as one of the major limitations to realizing the benefits of investment in health research. The gap between evidence and application warrants a greater investment in understanding how to advance the application of currently known efficacious treatments [1–3].

‘Knowledge translation’ (KT) is the term used in Canada to capture concepts such as implementation sciences, knowledge mobilization, diffusion, and research utilization [4, 5]. The KT field examines how to make research results relevant and applicable to the individuals who are in a position to use that information to make changes in policy or practice, and investigates strategies to facilitate the adoption process. KT is a growing area of research that aims to fill the gap between knowledge and practice.

The Canadian Institute of Health Research (CIHR) has identified KT as a critical component of health research in Canada [5]*.* KT is under-studied and under-resourced, and is often the rate limiting step to realizing the benefits of health research. Stemming from KT is *integrated knowledge translation* (IKT). In Canada, several national funders encourage an IKT approach which applies the principles of KT to the entire research process from research design to analysis to implementation of findings. IKT has its roots in the ‘Mode 2’ research approach [6] with the aim of producing research that is more directly relevant to and applied by knowledge users [7, 8]. CIHR defines a *knowledge user* as “an individual who is likely to be able to use research results to make informed decisions about health policies, programs and/or practices” [5]. Key to IKT is the partnering of researchers with knowledge users, often including knowledge users as members of the research team.

A knowledge user’s level of engagement in the research process may vary in intensity and complexity depending on the nature of the research and on the knowledge user’s needs [9]. Ross et al. studied researcher and decision maker collaboration in seven funded research programs [9]. Based on interviews with researchers and decision makers, they posited three different models of decision maker involvement: formal supporter, responsive partner, and integral partner. They suggested that each model might be appropriate given the specific needs of the research program. Sibbald et al. conducted a mixed method study of research studies funded by CIHR. They proposed different types of partnerships which included token, asymmetric, and egalitarian with the latter role consisting of an equal partnership and a symbiotic relationship between the researchers and knowledge users [10].

Other collaborative models have the goal of making research more relevant and responsive to an organization’s needs. Researchers in the United Kingdom have described embedded researchers or ‘researchers in residence’ as a model for co-production of research [11–13]. In this model, the researcher has a role within the organization, research is produced collaboratively, and is considered to be ‘jointly-owned’ [12]. Vindrola-Padros and colleagues (2018) posit that relationship building is central in an embedded researcher model which enables the researcher to understand the organizational context [13]. Similarly, the linkage and exchange model of knowledge brokers requires that strong relationships be developed between researchers and organizational decision makers [14]. Unlike the IKT approach, knowledge brokers are not usually members of the research team for a given project. Instead, a recent systematic review described knowledge broker roles as knowledge managers, linkage agents, and capacity builders [15].

Recently, a body of work has emerged which seeks to identify and address barriers and successes to IKT activities. According to Straus, Tetroe, and Graham (2009) key factors that make researcher - knowledge user collaborations successful are: (i) developing a shared understanding and common language about the issue or problem; (ii) creating a collaboration plan that involves the explicit description of researcher and knowledge user roles and responsibilities on an ongoing basis; (iii) creating a plan for including team members in a collaborative manner; (iv) developing a strategy to ensure trust amongst members of the collaboration and a plan to solve conflict as it arises. These authors also note that institutional support in both academic and knowledge user environments can facilitate the IKT process and ensure its success [16]. Grunfeld et al. (2004) suggest key points in the research process where knowledge user collaboration is particularly important: defining the research question and methodology; carrying out the methods; publishing findings; contextualizing findings and making changes (i.e. practice or policy) based on findings; and influencing future research projects [17]. Contextual factors may affect the success or failure of IKT. These factors include the circumstances under which research takes place (including collaborators and partnerships with organizations), relationships between and within the research team and knowledge users, the degree to which researchers and knowledge users understand IKT, and organizational pressures and priorities. Gagliardi et al. suggest that barriers to knowledge exchange between researchers and knowledge users include lack of leadership to promote knowledge exchange opportunities, lack of time, lack of regular opportunities for interaction, resistance to change, insufficient resources and lack of incentives [18].

The current study aimed to provide greater insight into contextual enablers and barriers to IKT. The specific study objectives were: (1) to learn how IKT activities were conducted within five projects of a cancer research network between 2010 and 2013; (2) to identify key IKT activities deemed to be either successful or unsuccessful by researchers and knowledge users; and (3) to identify and describe the key contextual factors that led to either successful or unsuccessful IKT activities. We used the term ‘knowledge user’ to indicate any member of a research team who could implement findings in health care practice and/or cancer system policy.

## Methods

### Multiple embedded case study

We used a multiple embedded descriptive case study design [19]. Each case was one of five studies within an overarching cancer health services research network (https://oicr.on.ca/research-portfolio/health-services-research/). The five studies addressed one of the following topics: case costing of cancer, lung cancer surgery policy analysis, patient and provider reported outcomes, colorectal cancer screening, and an inter-disciplinary team approach to women’s cancer survivorship (Table 1). The duration of each study was approximately 5 years. The cancer health services research network encouraged researchers to take an IKT approach in the design of their proposal. An IKT approach was viewed positively but it was not a requirement of funding.

**Table 1:** Description of cases within cancer health services research network and their study objectives

Case	Study objectives
1. Case costing of cancer	1. Review case costing literature and programs using administrative databases. 2. Determine availability and quality of cost data in administrative databases in Ontario. 3. Test a case costing methodology using different patient cohorts (e.g., disease sites). 4. Determine disaggregated costs. 5. Validate the resources and costs extracted from administrative linkages. 6. Create a report on the cost of cancer in Ontario and other publications.
2. Lung cancer surgery policy analysis	1. Describe the trends in the distribution of lung cancer surgery in Ontario between April 2003 and March 2009. 2. Estimate the effect of the policy on surgical outcomes. 3. Analyze how structures and processes of care have been affected by changes in the organization and delivery of lung cancer surgery. 4. Explore how a policy to regionalize lung cancer surgery in Ontario was perceived by healthcare decision-makers, healthcare providers, and patients who received lung cancer surgery.
3. Patient and provider reported outcomes	1. To perform linkages between these symptom datasets and administrative data, thereby potentially creating an unparalleled cancer outcomes database. 2. To evaluate whether formal symptom assessment in the course of routine clinical care might be used to predict ER utilization. There were three other specific research aims (not included).
4. Colorectal cancer screening	1. To evaluate the proposed mailed invitations prior to dissemination. 2. To describe the perceptions of the recipients of mailed invitations regarding: screening to prevent CRC; the mailed invitation; and their screening experiences following receipt of the invitation. 3. To describe the perceptions of participating PCPs with respect to: a) the ColonCancerCheck program; b) the mailed invitation; and c) the Screening Performance Report. 4. To describe the proportion, characteristics and outcomes of eligible Ontarians who responded/did not respond to the mailed invitations.
5. Inter-disciplinary team approach to women’s cancer survivorship	1. Create an inter-disciplinary team to address clinically important research questions related to the interplay between cancer and other medical conditions using the best data and analytical methods. 2. Create a cadre of researchers with interest and expertise in cancer survivorship research through graduate education, supervision and mentorship of trainees. 3. Ensure that the results of the research have a positive effect on clinical practice and patient outcomes through the development of an integrated knowledge translation strategy.

Case studies are ideal for examining the phenomenon of interest together with contextual issues [19]. Flyvbjerg has suggested that the value of case studies is that they are context-dependent [20]. Our justification for using a case study design is that it allowed us to explore the nuances and complexities of the projects within a cancer research network, including the similarities and differences amongst them [21]. In the current study, we applied IKT principles [5, 22–28] whereby stakeholders from the cancer health services research network including those with a formal role in the cancer system organization took part in all aspects of the study from forming the research questions through to interpretation of the findings, and potential implementation. We hypothesized that the results of this study would provide insights about IKT within a cancer health services research network that could be applied to the future research projects, and more broadly within the field of cancer research.

### Sampling

The sampling frame for the cases consisted of all five studies funded as part of the cancer health services research network between 2010 and 2013. The aim of the network was “to inform health policies, optimize the delivery of cancer care and maximize the benefits of the province’s cancer research for those currently living with the disease.” [https://oicr.on.ca/research-portfolio/health-services-research/]. In the current study, we aimed to recruit one or more researchers from each case and their respective knowledge users. Researchers on each of the five projects were identified by having been named on a publicly available summary of the cancer health services network. The knowledge users were identified by reviewing each project’s proposal and looking for descriptors in the proposal including the terms ‘knowledge user’ or ‘decision-maker’. In addition, we examined the affiliations of each team member for indications that they may have had decision-making responsibilities. The identity of the knowledge users was confirmed during interviews with researchers. Snowball sampling [29] led to other participants who were identified by researchers and knowledge users as being involved in the projects.

### Recruitment

Participants were contacted via email and invited to participate. Follow-up contact was completed by a research assistant (AC). Participants were contacted up to three times.

### Data collection

#### Document review

We conducted a document review of proposals, reports, and publications from each case. Information about the study objectives, methods and context was extracted. We also extracted direct quotes from the proposal for each case looking for evidence of IKT [5] (e.g., knowledge user involvement in the development of the study protocol, obtaining knowledge user suggestions or expertise on how the study should proceed, interpretation of the results, implementation of the results). We then looked for similarities and differences across the cases in descriptions (or lack thereof) of IKT including participants and formats. The document review also provided us with a preliminary list of key informants to contact for interviews. MS Excel was used to store information. All data were extracted by one team member (AC) and checked by another researcher (MAO). Discrepancies were minimal and were resolved by reviewing the source document.

#### Semi-structured interviews

Interview guides were developed and pilot-tested. For researchers, the interview guide focused on their perceptions of IKT within their studies, successes and challenges to taking an IKT approach, and recommendations for improving IKT approaches within the cancer health services research network that supported their research project. For knowledge users, the interview guide focused on perceptions of and satisfaction with their role as knowledge users on the projects, facilitators and barriers to their involvement, areas where an IKT approach could have been improved, and recommendations for improving IKT. All interviews were conducted by telephone by the research assistant (AC) who had expertise in qualitative methods.

### Coding and data analysis

Interviews were audio-recorded and transcribed. Personal identifiers were removed from the transcribed data then de-identified data were imported into QSR NVivo (Version 9). The analytic process was guided by principles of the constant comparative method [30]. We used descriptive coding for both interview and document data. Descriptive coding is a common technique in qualitative research. It is used to summarize in phrases the basic idea of passage of data, which contains the content of the message. Thus, the descriptive code captures the topic of the passage, while the passage itself contains the substance [31]. To ensure consistency of codes and emerging themes, two researchers independently compared all newly collected data with those collected earlier in the study. Once all interviews were coded, we integrated the interview and document review data. Thematic analysis was used to analyze the data set [32]. Thematic analysis is used to identify, analyze, and report patterns and/or themes within a particular data set. During the analysis, we first examined the data of each case (within case analysis), and then compared data across cases (cross case analysis). The results of the analysis were reviewed with the broader research team. Rigor in the analytic process was addressed by documenting analytic decisions in memos, having two research assistants independently code interview transcripts and collecting data from documents and from interviews.

### Research ethics board review

All participants provided written informed consent to participate in the study. The University of Toronto Research Ethics Board approved this study - Protocol reference # 32306.

## Results

Of the 25 participants who were contacted, 19 responded and 18 were interviewed. One respondent (a knowledge user) indicated that they did not have enough information to contribute to an interview as they perceived they were minimally involved in the project. Another respondent referred our team to a delegate to be interviewed in their place as the nominated individual was involved in the implementation of that particular project. We subsequently interviewed the nominated delegate. One researcher suggested we also contact another research team member; this person agreed to be interviewed. Researchers from one case did not respond to our invitation; a document review was completed but knowledge users from that case were not contacted for an interview. In total, five researchers and 11 knowledge users (one to three per case) across four cases were interviewed, as well as two stakeholders who had oversight responsibilities within the cancer health services research network.

### Description of participants

On average, researchers were 45 years old; knowledge users were 52 years old. Of all 18 participants, 11 were female and seven were male. Four researchers reported having either a hospital or university affiliation and one reported an affiliation with the cancer system organization. One researcher indicated that they had an embedded role within the cancer system organization. Of the knowledge users, seven reported having an affiliation with the cancer system organization; the others reported having either a hospital or university affiliation.

### Within case analysis

#### Case 1

There was no description of an IKT plan in the proposal. None of the participants mentioned having an explicit discussion of knowledge user role on the project; however, one participant mentioned that they believed their role as knowledge user “was understood” and it did not need to be made explicit. The knowledge users on Case 1 acted more as advisors. They were engaged at the beginning of the project and at the end by providing feedback on results, but not during the design of the project. While the information generated in Case 1 was important according to both the researcher and knowledge users, the project was not perceived to align explicitly with the organization’s goals. There did not appear to be a clear plan for knowledge users to take the results to their organization. The participants in this case also reflected that the organization as a whole did not have a framework or processes for using research results. One knowledge user indicated that the cancer system organization has had challenges in how it “integrates research and researchers into what it sees as its mission.” Another said,

“…I think that [cancer system organization] needs a really robust integrated KT strategy which I don’t recall has ever actually been implemented.” (Knowledge User)

The researcher of this case confirmed this view by saying that the organization did not have a “formal KT framework in place”. The researcher also indicated that in a large organization, it may be necessary to have knowledge users at different levels in the organization including leadership and management. Another insight generated by this case was that the organization employed people to conduct similar work as that undertaken by the research study. The researcher commented that there needed to be clear links between internal work by the organization and research activities by researchers who may be external to the organization.

#### Case 2

Although there was an IKT plan in the proposal, some aspects of the proposed IKT plan did not occur. For example, it was proposed that the knowledge users would be involved in the study design phase and in the interpretation of findings, but according to the knowledge users, this involvement did not occur. Knowledge users indicated that they were minimally engaged by the research team throughout and that they would have liked more input on the project.

For example,

“I would say that it [expectation for role] has not [been met] in the sense that I might have thought that there would be a somewhat higher level of engagement…” (Knowledge User)

The researcher expressed that there was some tension during the project and that “ideally” researchers would be independent. There was no clear implementation strategy beyond generating knowledge. In discussions with the researcher, there was also internal work that had been conducted by the organization on the same topic. It was not clear how the results of the internal work related to the research project.

#### Case 3

There was an IKT plan in the proposal. Although the researcher indicated that the research team was largely responsible for the research design, knowledge users said that they were also involved in the design phase of the project. Both researcher and knowledge users indicated that they were engaged throughout the duration of the project. However, the engagement of knowledge users appeared to be significantly impacted by their turnover in the organization. When asked about the knowledge users on the project, the researcher indicated that knowledge users who had started on the project had all left the organization. The following quotation illustrates the problem of turn-over within the cancer system organization,

“… [name of cancer system organization] is notorious for having high rates of staff turnover. Having continuity of person and kind of that memory you know across the life of the project can be a problem…You know it is not unique to me or to my program but… I see that quite a lot. You know people move, they change portfolios, they leave for other jobs, all sorts of other things happen. It is hard to have the same person doing the same thing for any stretch of time that you can build a meaningful relationship with them.” (Researcher)

The researcher indicated that the relationship they had with a newer knowledge user on the project was “never quite as strong” as in the previous relationship. While knowledge users shared the results with their organization and other audiences, there did not appear to be a plan for implementing the results. According to the researcher, the results of the study were implemented in a quality index in the cancer system organization.

#### Case 4

There was an IKT plan in the proposal. While two other cases indicated that knowledge users were involved in the planning phase of those projects, Case 4 was somewhat different. In this case, the research team indicated that it developed the project alongside the knowledge users and their organization based upon pilot work that was ongoing at the organization. As a result, the research project was closely aligned with the ongoing needs and priorities of the organization, and the knowledge users were in a position to make changes based on results. The researcher and knowledge users indicated that knowledge users were engaged throughout the project and were involved in all aspects of the study. Knowledge users provided feedback on results and worked with the researcher to make programmatic changes within the relevant area of the cancer system. The researcher, who had an embedded role in the organization continued to work with knowledge users on implementation.

Table 2 provides details of knowledge user engagement for each case by study phase and includes exemplar quotations. Three of four cases included an IKT plan in their proposals. However, the degree to which the proposed IKT plan was realized, and the engagement of knowledge users at all phases of research, varied. The participants of Cases 1 and 2 seemed to have limited knowledge of some of the principles of an IKT approach. In Case 3, there were IKT efforts; however, these seemed to be more directed by the researcher and knowledge user turnover was perceived to hinder IKT activities. Case 4 appeared to be most closely aligned with principles of IKT whereby there was engagement of knowledge users at all phases of research, knowledge users held positions within the organization in which they could act on the results, and there was a close alignment of the project with organizational priorities.

**Table 2:** Knowledge user involvement in cancer health services research project by study phase and case

Study Phase	Case 1	Case 2	Case 3	Case 4
1. Involved in planning stages (i.e. defining research question, designing study)	Document Review (Proposal^a^): - Not described Interviews: - Researcher did not indicate that knowledge users (KUs) were involved in the planning stages. - 2/3 KUs indicated they provided access to data but were not involved in planning stages; 1/3 KUs indicated they were involved in study design - All 3 KUs indicated they had an advisory role.	Document Review (Proposal): - “The decision-maker partners will be engaged in the research throughout the project, including the development and refinement of the research questions…” (pp.66) Interviews: - Researcher indicated that KUs were involved in planning stages, but had a limited role - 2 KUs indicated they were involved in the planning stages. - 1 KU could not recall their involvement.	Document Review (Proposal): - “[Names of two KUs] have been involved in the development of this project and articulation of its goals…” (pp.93) - “They have provided input on key methodological issues in the drafting of this proposal…” (pp.93) Interviews: - Researcher indicated that the research team did most of the project design - 1 KU described themselves as an adviser - 2 KUs said that they were involved in the research design stage with researchers.	Document Review (Proposal): - “Key stakeholders have been involved in the planning stages…of this research project.” (pp.55) Interviews: - Researcher indicated that KUs were involved in planning stages, which was built from a pilot done by the cancer system organization. - KUs indicated that the cancer system organization was involved in the “front end” and research approach was developed collaboratively with researchers.
2. Involved in methods and/or analysis throughout the study	Document Review (Proposal): - Not described Interviews: - Researcher did not indicate that KUs were involved in methods or analysis throughout the study. Researcher indicated that they presented the results to KUs. - KUs confirmed the researcher’s views.	Document Review (Proposal): - “The entire study team will meet every 3 months to review analytic plans, interpret findings, and assign research tasks. The decision-maker partners will be engaged in the research throughout the project, including the development and refinement of the research questions, study methodology, and development of instruments and measures.” (pp.66) Interviews: - Researcher did not indicate KU involvement in methods or analysis - 2 KUs indicated there were minimal meetings and informal updates when the research was being conducted; - 1 KU did not recall their involvement.	Document Review (Proposal): - Not described Interviews: - Research team held regular meetings to keep KUs involved and informed throughout the project, and to obtain feedback from KUs. - 1 KU indicated they provided input throughout the project, and that there were regular meetings and email communication. - 1 KU indicated they were engaged “all the way along” - 1 KU left their organization role.	Document Review (Proposal): - “A working group comprised of [*description of KUs*] and study investigators will meet regularly throughout the project to ensure continued integrated KT [knowledge translation].” (pp.55) Interviews: - Researcher indicated one KU was involved in methods - 1 KU said they were engaged but had a role change and could no longer continue as a KU, but researcher kept them informed about study activities.
3. Provided feedback on results	Document Review Proposal: -“To encourage this exchange, we will convene an advisory panel made up of representatives … to communicate findings and discuss implications of the data derived which may also lead to further sub-investigations.” (pp.101–102) Interviews: - Researcher and KUs indicated that, as advisers, they provided feedback on data and findings	Document Review Proposal: - “Regular reporting of results to the team will ensure timely progress and adjustments for further analyses and ultimately will result in findings which are relevant to the decision-makers.” (pp.66) Interviews: - Researcher indicated that more KU involvement came at the point when results were being reviewed. - 2 KUs indicated they provided feedback on findings - 1 KU did not recall their involvement.	Document Review Proposal: - “[KUs] will participate in reviewing and interpreting results of analysis.” (pp.93) Interviews: - Researcher indicated that KUs provided feedback on results and suggested future research directions. - 2 KUs indicated they provided feedback on results - 1 KU indicated that results were not available when they part of the project.	Document Review Proposal: - “The findings from each aim will be reviewed, analyzed and interpreted with input from this working group.” (pp.55) Interviews: - Researcher indicated KUs provided feedback on results then KUs used those findings to make programmatic changes. - 1 KU left before findings were available - 1 KU joined project to implement results once project was finished.
4. a) Shared results with cancer system organization	Document Review Proposal: - Not described Interviews: - Researcher indicated that the research team shared results cancer system organization - 1 KU did not recall discussing results with organization. They were not sure if research team had sent them the results. - 2 KUs did not recall discuss results with the organization.	Document Review Proposal: - Not described Interviews: - Researcher was not aware if KUs had shared the results of the project with their organization - 1 KU indicated that they presented results at a cancer system organization meeting - 1 KU did not indicate they shared results with their organization - 1 KU did not recall their involvement.	Document Review Proposal: - Not described Interviews: - Researcher team was invited to cancer system organization to present results. - 1 KU indicated that they discussed project results with “lots of people” at the organization. - 2/3 KUs did not indicate that they shared results with organization	Document Review Proposal: - “The findings from each aim will be reviewed, analyzed and interpreted with input [from organization]…key findings will be communicated to other stakeholders integral to the conduct of the [name] program.” (pp.55) Interviews: - Researcher indicated that the team wrote a report for the KUs and the KUs used it to make program changes at cancer system organization. - 1 KU left role so was not involved in any sharing of results to cancer system organization - 1 KU joined project as a decision maker to implement results once project was finished.
4. b) Shared results with other audiences	Document Review Proposal: - Not described Interviews: - Neither researcher nor KUs indicated that KUs shared results to audiences outside of cancer system organization.	Document Review Proposal: - Not described Interviews: - Researcher did not indicate that KUs had shared the results of the project with other audiences. - None of the KUs indicated that they shared the results with other audiences outside of organization.	Document Review Proposal - Not described Interviews: - 1 KU indicated that they shared the results to clinical audiences nationally and internationally - 1 KU changed roles during project but says they still reference the results in different presentations to other audiences outside of organization.	Document Review Proposal: - Not described Interviews: - Neither researcher nor KUs indicated that KUs shared results to audiences outside of organization.
5. Implemented study results	Document Review Proposal (plan to implement): - Not described Interviews: - Researcher indicated that they are currently determining how to implement results and aligning with cancer system organization priorities. - None of the KUs said that they implemented results.	Document Review Proposal (plan to implement): - Not described Interviews: - Researcher did not indicate that KUs implemented the results. - 1 KU indicated that the researcher did not give them direction or tell them what to do next. - 1 KU was not aware if results were used by cancer system organization. - 1 KU did not recall their involvement.	Document Review Proposal (plan to implement): - Not described Interviews: - Researcher did not indicate that KUs implemented results, but continued to work with KUs and the collaboration led to additional research questions and projects. - 1 KU said they used the results to build a case around the validity of cancer system work and changes that should be made at a ‘clinical level’. - 1 KU left their role at cancer system organization; when they left, the KUs were working through how to implement results. - 1 KU left project and did not have a role in implementation.	Document Review Proposal (plan to implement): - “The objectives of our knowledge strategy are to improve [cancer system area] by: …translating the outcomes of our project to the broader research community.” (pp.55) - “With [KU] assistance, key findings will be communicated to other stakeholders integral to the conduct of the [cancer system organization program].” (pp.55) Interviews: - Researcher indicated that results were presented to KUs, which informed future cancer system organization activities, changes, and further research designs. - 1 KU indicated that program changes were made, but they themselves did not use results because they left the organization - 1 KU works with researcher often, and KU’s cancer system organization used project results to make cancer system process changes.

^a^Refers to plans to involve knowledge users in various phases of project

### Cross case analysis

We identified features of research projects that participants perceived to be successful and/or unsuccessful in encouraging collaboration between researchers and knowledge users. These features are summarized in Tables 3 and 4 respectively.

**Table 3:** Researchers and knowledge users’ views on factors that encouraged knowledge user collaboration on cancer health services research projects

Factor	Notes
1. Research team discusses roles at beginning of project^a^	Participants recommended that in future health services research network projects an explicit discussion of researcher and knowledge user (KU) roles take place at the beginning of the project.
2. KUs have decisional authority within the organization to implement project results^a^	While KU decisional authority was identified as important, not all KUs had such authority within their organization.
3. Researchers engage KUs throughout duration of project (e.g., via regular contact, checking in, meetings)^a^	KUs preferred to be engaged throughout the study in contrast to collaborations where KUs are minimally engaged or engaged at the beginning and/or end of the project, but less throughout. No discussion by researchers or KUs that KUs would take initiative to engage with researchers throughout the project.
4. Project goals align with organizational goals and priorities^a^	Project goals may be important but they must align with the organization’s goals and priorities for results to be considered for implementation by the cancer system organization.

^a^Identified in all cases

**Table 4:** Researcher and knowledge users’ views on factors that discouraged knowledge user collaboration on cancer health services research projects

Factors	Notes
1. No explicit discussion of knowledge user (KU) role^a^	Lack of an explicit discussion of roles may have led to ambiguity about KU role throughout the project including their role in sharing results in the KU’s organization.
2. KUs do not have decisional authority within the organization to implement project results^a^	Not all KUs had decisional authority to implement the results at the organization.
3. No clear implementation plan for results in KU’s organization^b^	The goal of specific projects may have been to generate new knowledge, but there was no plan as to what exactly would be done with that knowledge in the KU’s organization.
4. Lack of alignment between project goals and organizational goals and priorities^b^	Project goals must align with the organization’s goals and priorities for results to be considered for implementation by the cancer system organization.
5. Lack of time for KU involvement in project and/or geographical distance from researchers^a^	KUs had multiple responsibilities and had to make time for involvement in project. Being a KU on a project was not part of their usual role in the organization. In some projects, KUs were located in another city and interactions occurred by teleconference.
6. KU turnover at organization^a^	KU turnover was perceived to significantly impact collaboration on projects. For example, project history was lost when a new KU joined the study team.
7. Lack of knowledge translation framework or process in KU’s organization^b^	KUs commented that without a knowledge translation framework within the organization, it was difficult to share results of research study with key decision-makers. While the KUs used the term “framework”, it is possible that other organizational features such as knowledge translation structures and processes may be needed.

^a^Identified in all cases

^b^Identified in 2 cases

Although the context of each case was unique, there were similarities across all four cases. First, participants indicated that there was no explicit discussion about research team or knowledge user roles, although this was not necessarily seen as a problem for all participants. For example, one knowledge user indicated that an explicit discussion with the researcher about their role was not needed as the knowledge user had been involved in research before and had assumptions about their role that did not need to be expressed. Second, in all cases, participants acknowledged that the role of knowledge users was to provide access to data or expertise, and to make research results more relevant to their organizations. However, in three of four cases, the degree to which the knowledge users had the ability to act upon the results or the degree to which there was an implementation plan varied. Third, across all four cases, time and distance were perceived as challenges to knowledge user participation. Knowledge users had to manage their schedules to include collaboration with researchers, which went above and beyond their regular roles. Lastly, while Case 3 experienced more knowledge user role turnover during the project, participants across all four cases identified knowledge user turnover within the organization as a problem for researcher-knowledge user collaboration. This was acknowledged as a difficult issue to resolve given that academic research projects typically take several years to complete, and researchers cannot control whether a knowledge user leaves their organization or leaves the research team because their role within the organization changed.

In two of four cases, perceived uptake of results with the cancer system organization appeared to be related to having an IKT approach in the study. In both cases, the organization was already engaged in the topic area and acknowledged the importance of the project and its results. In another case, the cancer system organization was also invested in the topic area. Although the goal of the research project was to evaluate the implementation of a policy, the knowledge users expressed that they did not know how to use the study results. In the last case, there was no evidence that there was uptake of the results within the organization.

## Discussion

The results of the current study indicate that there is a need for elucidation of the principles of IKT and the roles of both knowledge users and researchers on cancer-related research studies. First, based on document review and interview data, we can speculate that in some cases, researchers and knowledge users did not fully understand the principles of IKT. Second, the majority of participants in three of the four cases, expressed that the roles of knowledge users could have been made more explicit. We suggest that if an IKT approach is desired, all collaborators play an equal role in defining roles and making expectations explicit from the beginning of the research project [33–36].

Another finding from the current study was that both researchers and knowledge users perceived that the cancer system organization lacked a framework for an IKT approach and an ‘intake’ process for research results. For cancer system organizations to be able to promote evidence-based care, it seems obvious that there needs to be an explicit process for how results from research studies can be considered for action by the organization.

Our study has identified the need for clarification on the role of knowledge users on research studies. For several of the knowledge users, their role on the study was not part of their usual responsibilities within the organization and they had to make time for this extra role. This is especially problematic if the knowledge user left the organization, taking the project history with them. Given that knowledge user involvement on research collaboration was not part of their organizational roles, it is understandable that IKT efforts were largely led by the research team. We suggest that collaboration on research projects be explicitly part of the knowledge user’s role so that if a new person takes over the role, the project history is not lost. This assumes buy-in from the organization that a knowledge user role is important to the work of the organization and there is an explicit hand-over from one knowledge user to the next.

Our results share some similarities with the findings of other researchers. Ross et al. [9] described different models of decision-maker involvement in research, specifically formal support, responsive partner, and integral partner. In our study, we found that the roles of knowledge users on two of the cases were as “advisors” who were supportive of the goals of the research project and facilitated access to data. This advisor role seems similar to the role of formal supporter described by Ross. Several of the participants in our study also indicated that was important to engage the “right level” of person in the organization. We believe this finding to be an important insight. While a knowledge user may be sufficiently engaged throughout all phases of a research project, if that knowledge user does not have decision-making authority in their organization, the IKT efforts may fall short. In a large and complex organization, different knowledge user roles may be needed if research results are to be considered for implementation. These various roles could include knowledge users who would sanction implementation at the policy level, knowledge mobilizers who would hold managerial roles, and knowledge implementers who would be responsible for implementing the results within the organization.

Several of our findings about facilitators of IKT are similar to benefits of an embedded researcher model [12, 13]. These include the importance of developing relationships between researchers and decision makers within the organization and ensuring that the proposed research is responsive to organizational needs and priorities. However, in the current study, we investigated participants’ perceptions of an IKT approach rather than an embedded researcher model. Only one of the researchers in our cases described themselves as having an ‘embedded’ role in the organization; the others indicated an academic or hospital affiliation. We speculate that an embedded researcher model may be one strategy for facilitating an IKT approach but this presumption would require further investigation.

Our study findings support the work of Gagliardi and Dobrow [37] who derived a framework for assessing organizational capacity for IKT. In particular, the linkage of IKT to organizational priorities resonated with the findings of three of four cases in our study. At the individual level, we also found an apparent lack of knowledge of the principles of IKT among some researchers and knowledge users. We also found in one case that the researcher valued independence of the research from the knowledge users. Even if knowledge users are interested in being involved in a study, if the researchers value independence then it is difficult to imagine that the knowledge users would have a meaningful role in the research study.

A novel finding of the current study is about the perceived importance of internal work by the organization on the same topic as the one pursued by the researchers. It is possible that there may be less investment in external research and more valuing of internal research completed by employees although this finding needs further investigation.

Several of our findings were similar to those of a recent scoping review of IKT [38]. Specifically, Gagliardi reported nine barriers and 15 enablers of IKT. In our study we found six of the nine barriers reported by these researchers: differing needs and priorities, lack of skill in or understanding of IKT processes, lack of clarity in goals, roles, and expectations, little continuity of involvement due to staff turnover, limited time of knowledge users who had multiple responsibilities, and geographical distance. In addition, we found two other barriers: minimal engagement of knowledge users by researchers and lack of a clear implementation plan for the results. With respect to enablers, there were three of 15 enablers in common: immersion of researchers in the decision-maker setting, building on pre-existing relationships, and establishing partnership early in the research process. It is possible that differences between the results of the current study and those of the scoping review could have occurred because many of the knowledge users in our study were affiliated with the same cancer system organization and had similar experiences. It is possible that we did not find many enablers because of the limited numbers of cases in our study. Had we been able to sample other cases, it is possible that we might have found additional enablers.

A unique perspective offered by the current study is that we conducted a multiple case study of IKT using an IKT approach. Four of the co-investigators had a role in the cancer system organization and were involved in the design, interpretation of the findings, and drafting of the manuscript. However, we too have experienced difficulty in identifying the ‘right’ audience for our findings in a large organization. The knowledge users on our own study have all leadership roles but within different portfolios making it difficult to find a home when the findings have implications across the organization. An immediate benefit of the IKT approach was the case study results were used in the design of a subsequent funding application by the cancer health services network.

### Limitations

There are several limitations to our study. The interviews occurred approximately 2 years after the completion of the project. Individuals may not have recalled details of their involvement in the study. It is also possible that those who declined our invitation to participate would have had different perspectives compared to those who did participate.

It is possible that we could have missed a knowledge user from one or more of the cases. However, we sought knowledge users from the document review and used snowball sampling to locate other knowledge users.

We were limited to four cases all within the same cancer health services research network and as such were restricted in our ability to find confirming and disconfirming results across the cases. However, an advantage was that the cases were heterogeneous and included different topic areas and varying methodological approaches. This study focussed on cases within a cancer health services research network; we cannot comment on other instances of IKT internal to the cancer system organization.

## Conclusions

This case study provided detailed information about IKT plans and implementation within a cancer health services research network. Barriers to KU engagement in the co-production of cancer health services research include lack of knowledge of IKT principles amongst researchers and knowledge users, mismatched expectations of knowledge user role, frequent KU turnover within the organization, and a lack of a knowledge translation framework or processes within the organization. When a research study is directly aligned with organizational priorities, it appears more likely that results generated by research will be considered in programming. Research teams will need to consider specific strategies to address barriers to KU engagement.

## Abbreviations

IKT: Integrated knowledge translation
KT: Knowledge translation
KU: Knowledge user
